# 2-[3-(Naphthalen-2-yl)phen­yl]naph­thal­ene^1^

**DOI:** 10.1107/S1600536813002390

**Published:** 2013-01-31

**Authors:** Mark L. Wolfenden, Raj K. Dhar, Frank R. Fronczek, Steven F. Watkins

**Affiliations:** a Department of Chemistry, Louisiana State University, Baton Rouge LA, 70803-1804, USA

## Abstract

The title compound, C_26_H_18_, consists of a benzene ring with *meta*-substituted 2-naphthalene substituents, which are essentially planar [r.m.s. deviations = 0.022 (1) and 0.003 (1) Å]. The conformation is *syn*, with equivalent torsion angles about the benzene–naphthalene bonds of −36.04 (13) and +34.14 (13)°. The mol­ecule has quasi-*C*_s_ mol­ecular symmetry.

## Related literature

For properties of oligophenyls, see: Bocchinfuso *et al.* (2009 ▶) and for their synthesis, see: Marcinow & Rabideau (1990 ▶); Du *et al.* (1986 ▶); Woods *et al.* (1951 ▶). For similar structures, see: Baker *et al.* (1990 ▶); Lin & Williams (1975 ▶); Bart (1968 ▶); Tummala *et al.* (2013 ▶). For conformational calculations with *GAUSSIAN09*, see: Frisch *et al.* (2009 ▶).
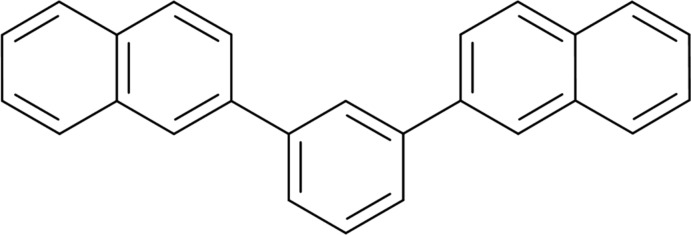


## Experimental

### 

#### Crystal data

C_26_H_18_
*M* *_r_* = 330.4
Orthorhombic, 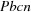

*a* = 25.9304 (3) Å
*b* = 8.9300 (1) Å
*c* = 14.9377 (2) Å
*V* = 3458.95 (7) Å^3^
*Z* = 8
Mo *K*α radiation
μ = 0.07 mm^−1^
*T* = 100 K
0.40 × 0.27 × 0.22 mm

#### Data collection

Nonius KappaCCD diffractometer
Absorption correction: multi-scan (*SCALEPACK*; Otwinowski & Minor, 1997 ▶) *T*_min_ = 0.972, *T*_max_ = 0.984
37425 measured reflections
6240 independent reflections
4993 reflections with *I* > 2σ(*I*)
*R*_int_ = 0.023

#### Refinement

*R*[*F* ^2^ > 2σ(*F* ^2^)] = 0.045
*wR*(*F* ^2^) = 0.125
*S* = 1.02
6240 reflections
289 parameters
Only H-atom coordinates refined
Δρ_max_ = 0.38 e Å^−3^
Δρ_min_ = −0.22 e Å^−3^

### 

Data collection: *COLLECT* (Nonius 2000 ▶); cell refinement: *SCALEPACK* (Otwinowski & Minor, 1997 ▶); data reduction: *DENZO* (Otwinowski & Minor, 1997 ▶) and *SCALEPACK*; program(s) used to solve structure: *SIR2002* (Burla *et al.*, 2003 ▶); program(s) used to refine structure: *SHELXL97* (Sheldrick, 2008 ▶); molecular graphics: *ORTEP-3 for Windows* (Farrugia, 2012 ▶); software used to prepare material for publication: *WinGX* (Farrugia, 2012 ▶).

## Supplementary Material

Crystal structure: contains datablock(s) global, I. DOI: 10.1107/S1600536813002390/pv2619sup1.cif

Structure factors: contains datablock(s) I. DOI: 10.1107/S1600536813002390/pv2619Isup2.hkl

Supplementary material file. DOI: 10.1107/S1600536813002390/pv2619Isup3.cml

Additional supplementary materials:  crystallographic information; 3D view; checkCIF report

## Figures and Tables

**Fig. 1.**
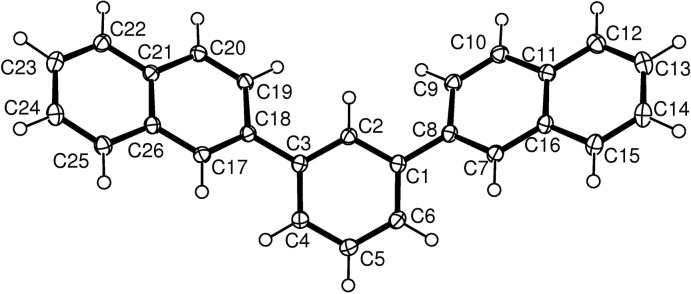
View of (I) (50% probability displacement ellipsoids)

## supplementary crystallographic information

### Comment

The crystal and molecular structures of *p*-oligophenyls have been well investigated (Baker *et al.*, 1990, and references therein), but relatively few studies have appeared concerning the conformational preferences of *m*-oligophenyls. Two interesting papers were reported, one by Lin & Williams (1975) about the crystal structure of 1,3,5 triphenyl, which serves as a model for *m*-polyphenyls, showing substituted phenyl groups being twisted about the formal single bonds by +40.7, -37.2, and +36.1° out of the plane of the central ring. The crystal structure of one of the polymorphic forms of hexaphenyl benzene, reported by Bart (1968), has shown that the peripheral rings are not perpendicular to the central ring, but are twisted by about 25°. The molecule was found to be highly distorted as a result of out-of-plane bending of the exocyclic bonds. Therefore, we have studied the structure of 1,3-bis(2-naphthyl)benzene for comparison of its conformation with the previous results.

Title compound I is of quasi-C_s_ symmetry and consists of a benzene ring with *meta*-substituted 2-naphthalenes. The benzene ring is nearly planar (C-atoms only, δ_r.m.s._ = 0.005 (1) Å), as are the two naphthalenes (δ_r.m.s._ = 0.022 (1) and 0.003 (1) Å). The benzene plane and both naphthalene planes are bent with respect to the benzene-naphthalene (BN) bonds: C8 and C18 lie above the benzene plane by 0.039 (2) and 0.040 (1) Å respectively, while C1 lies above its proximate naphthalene plane by 0.016 (1) Å, and C3 lies above its proximate naphthalene plane by 0.085 (1) Å. The naphthalene ring planes are also twisted about the BN bonds with equivalent torsion angles of -36.04 (13)° (C6–C1–C8–C7) and +34.14 (13)° (C4–C3–C18–C17). An isolated and optimized C_s_ model (Gaussian09; Frisch *et al.*, 2009; DFT:b3lyp/3–21 g) shows a small amount of bending about the BN bonds, with equivalent distances from mean planes C8/C18 = 0.020 Å and C1/C3 = 0.004 Å, and BN torsion angles of ± 43°.

### Experimental

Compound I was prepared after Du *et al.* (1986) and recrystallized from petroleum ether.

### Refinement

The positional parameters of all H atoms were refined, but *U*_iso_(H) was set to 1.2*U*_eq_ of the attached C atom. The range of C–H distances is 0.959 (14) - 1.020 (14) Å.

### Crystal data

**Table:**

C_26_H_18_	*D*_x_ = 1.269 Mg m^−^^3^
*M**_r_* = 330.4	Melting point: 143.5(5) K
Orthorhombic, *P**b**c**n*	Mo *K*α radiation, λ = 0.71073 Å
Hall symbol: -P 2n 2ab	Cell parameters from 6568 reflections
*a* = 25.9304 (3) Å	θ = 2.6–32.6°
*b* = 8.9300 (1) Å	µ = 0.07 mm^−^^1^
*c* = 14.9377 (2) Å	*T* = 100 K
*V* = 3458.95 (7) Å^3^	Prism, colourless
*Z* = 8	0.40 × 0.27 × 0.22 mm
*F*(000) = 1392	

### Data collection

**Table:**

Nonius KappaCCD diffractometer	6240 independent reflections
Radiation source: fine-focus sealed tube	4993 reflections with *I* > 2σ(*I*)
Graphite monochromator	*R*_int_ = 0.023
Detector resolution: 9 pixels mm^-1^	θ_max_ = 32.6°, θ_min_ = 2.7°
φ and ω scans	*h* = −39→39
Absorption correction: multi-scan (*SCALEPACK*; Otwinowski & Minor, 1997)	*k* = −13→13
*T*_min_ = 0.972, *T*_max_ = 0.984	*l* = −22→22
37425 measured reflections	

### Refinement

**Table:**

Refinement on *F*^2^	Primary atom site location: structure-invariant direct methods
Least-squares matrix: full	Secondary atom site location: difference Fourier map
*R*[*F*^2^ > 2σ(*F*^2^)] = 0.045	Hydrogen site location: inferred from neighbouring sites
*wR*(*F*^2^) = 0.125	Only H-atom coordinates refined
*S* = 1.02	*w* = 1/[σ^2^(*F*_o_^2^) + (0.0658*P*)^2^ + 0.9158*P*] where *P* = (*F*_o_^2^ + 2*F*_c_^2^)/3
6240 reflections	(Δ/σ)_max_ = 0.001
289 parameters	Δρ_max_ = 0.38 e Å^−^^3^
0 restraints	Δρ_min_ = −0.22 e Å^−^^3^
0 constraints	

### Special details

**Table:**

Geometry. All e.s.d.'s (except the e.s.d. in the dihedral angle between two l.s. planes) are estimated using the full covariance matrix. The cell e.s.d.'s are taken into account individually in the estimation of e.s.d.'s in distances, angles and torsion angles; correlations between e.s.d.'s in cell parameters are only used when they are defined by crystal symmetry. An approximate (isotropic) treatment of cell e.s.d.'s is used for estimating e.s.d.'s involving l.s. planes.

### Fractional atomic coordinates and isotropic or equivalent isotropic displacement parameters (Å^2^)

**Table:**

	*x*	*y*	*z*	*U*_iso_*/*U*_eq_	
C1	0.63414 (3)	0.65124 (10)	0.39203 (6)	0.01689 (17)	
C2	0.62992 (3)	0.71392 (10)	0.30628 (6)	0.01694 (16)	
H2	0.6484 (5)	0.8056 (15)	0.2934 (8)	0.02*	
C3	0.59837 (3)	0.65068 (10)	0.24032 (6)	0.01660 (16)	
C4	0.57139 (4)	0.51927 (11)	0.26073 (6)	0.01896 (17)	
H4	0.5494 (5)	0.4704 (15)	0.2136 (8)	0.023*	
C5	0.57577 (4)	0.45461 (11)	0.34519 (6)	0.02072 (18)	
H5	0.5574 (5)	0.3638 (16)	0.3575 (8)	0.025*	
C6	0.60671 (4)	0.51962 (11)	0.41055 (6)	0.01975 (18)	
H6	0.6101 (5)	0.4717 (15)	0.4700 (9)	0.024*	
C7	0.65216 (4)	0.72652 (11)	0.55034 (6)	0.01811 (17)	
H7	0.6193 (5)	0.6799 (15)	0.5698 (8)	0.022*	
C8	0.66657 (3)	0.72466 (10)	0.46121 (6)	0.01701 (16)	
C9	0.71347 (4)	0.79654 (11)	0.43622 (6)	0.01953 (17)	
H9	0.7242 (5)	0.7931 (15)	0.3721 (8)	0.023*	
C10	0.74404 (4)	0.86589 (11)	0.49847 (6)	0.02031 (18)	
H10	0.7772 (5)	0.9160 (15)	0.4807 (8)	0.024*	
C11	0.72952 (4)	0.86948 (10)	0.59028 (6)	0.01801 (17)	
C12	0.76011 (4)	0.94138 (11)	0.65616 (7)	0.02206 (19)	
H12	0.7930 (5)	0.9933 (16)	0.6373 (9)	0.026*	
C13	0.74530 (4)	0.94171 (12)	0.74436 (7)	0.0246 (2)	
H13	0.7680 (5)	0.9909 (16)	0.7903 (9)	0.03*	
C14	0.69902 (4)	0.87049 (13)	0.77044 (6)	0.0248 (2)	
H14	0.6888 (5)	0.8669 (17)	0.8349 (9)	0.03*	
C15	0.66831 (4)	0.80109 (12)	0.70813 (6)	0.02236 (19)	
H15	0.6365 (5)	0.7517 (16)	0.7256 (9)	0.027*	
C16	0.68280 (4)	0.79842 (10)	0.61631 (6)	0.01787 (17)	
C17	0.54718 (3)	0.72005 (10)	0.10483 (6)	0.01746 (17)	
H17	0.5158 (5)	0.6693 (15)	0.1293 (8)	0.021*	
C18	0.59332 (3)	0.72319 (10)	0.15125 (6)	0.01630 (16)	
C19	0.63624 (3)	0.79954 (11)	0.11308 (6)	0.01800 (17)	
H19	0.6705 (5)	0.8014 (15)	0.1453 (8)	0.022*	
C20	0.63221 (3)	0.86852 (11)	0.03141 (6)	0.01788 (17)	
H20	0.6622 (5)	0.9217 (15)	0.0055 (8)	0.021*	
C21	0.58490 (3)	0.86835 (10)	−0.01638 (6)	0.01625 (16)	
C22	0.57913 (4)	0.94470 (11)	−0.09892 (6)	0.01963 (18)	
H22	0.6099 (5)	0.9976 (15)	−0.1227 (9)	0.024*	
C23	0.53253 (4)	0.94754 (12)	−0.14213 (6)	0.02269 (19)	
H23	0.5289 (5)	1.0075 (16)	−0.1999 (9)	0.027*	
C24	0.48948 (4)	0.87247 (12)	−0.10533 (6)	0.02300 (19)	
H24	0.4556 (5)	0.8770 (16)	−0.1362 (9)	0.028*	
C25	0.49393 (4)	0.79656 (11)	−0.02576 (6)	0.02032 (18)	
H25	0.4636 (5)	0.7474 (16)	0.0014 (8)	0.024*	
C26	0.54175 (3)	0.79277 (10)	0.02090 (6)	0.01655 (16)	

### Atomic displacement parameters (Å^2^)

**Table:**

	*U*^11^	*U*^22^	*U*^33^	*U*^12^	*U*^13^	*U*^23^
C1	0.0171 (4)	0.0180 (4)	0.0155 (4)	0.0020 (3)	0.0003 (3)	−0.0006 (3)
C2	0.0186 (4)	0.0168 (4)	0.0154 (4)	0.0002 (3)	0.0008 (3)	0.0003 (3)
C3	0.0170 (4)	0.0177 (4)	0.0151 (3)	0.0020 (3)	0.0006 (3)	−0.0004 (3)
C4	0.0204 (4)	0.0185 (4)	0.0180 (4)	−0.0005 (3)	−0.0009 (3)	0.0001 (3)
C5	0.0237 (4)	0.0183 (4)	0.0202 (4)	−0.0023 (3)	−0.0001 (3)	0.0023 (3)
C6	0.0232 (4)	0.0189 (4)	0.0172 (4)	0.0007 (3)	0.0001 (3)	0.0028 (3)
C7	0.0170 (4)	0.0212 (4)	0.0161 (4)	−0.0001 (3)	−0.0001 (3)	0.0021 (3)
C8	0.0179 (4)	0.0172 (4)	0.0159 (4)	0.0016 (3)	−0.0009 (3)	0.0010 (3)
C9	0.0195 (4)	0.0220 (4)	0.0171 (4)	−0.0002 (3)	0.0018 (3)	0.0009 (3)
C10	0.0186 (4)	0.0221 (4)	0.0202 (4)	−0.0011 (3)	0.0012 (3)	0.0015 (3)
C11	0.0171 (4)	0.0182 (4)	0.0187 (4)	0.0019 (3)	−0.0011 (3)	0.0009 (3)
C12	0.0209 (4)	0.0212 (4)	0.0241 (4)	0.0006 (3)	−0.0031 (3)	−0.0015 (3)
C13	0.0251 (5)	0.0265 (5)	0.0224 (4)	0.0049 (4)	−0.0058 (4)	−0.0052 (4)
C14	0.0256 (5)	0.0319 (5)	0.0169 (4)	0.0058 (4)	−0.0019 (3)	−0.0024 (4)
C15	0.0218 (4)	0.0288 (5)	0.0165 (4)	0.0020 (4)	0.0005 (3)	0.0010 (3)
C16	0.0175 (4)	0.0205 (4)	0.0156 (4)	0.0022 (3)	−0.0013 (3)	0.0011 (3)
C17	0.0168 (4)	0.0187 (4)	0.0169 (4)	−0.0013 (3)	0.0012 (3)	0.0002 (3)
C18	0.0181 (4)	0.0161 (4)	0.0146 (3)	−0.0001 (3)	0.0004 (3)	−0.0010 (3)
C19	0.0161 (4)	0.0211 (4)	0.0168 (4)	−0.0011 (3)	−0.0007 (3)	−0.0007 (3)
C20	0.0175 (4)	0.0193 (4)	0.0168 (4)	−0.0024 (3)	0.0004 (3)	−0.0003 (3)
C21	0.0179 (4)	0.0157 (4)	0.0152 (4)	0.0001 (3)	0.0000 (3)	−0.0016 (3)
C22	0.0235 (4)	0.0189 (4)	0.0165 (4)	0.0001 (3)	0.0009 (3)	0.0011 (3)
C23	0.0271 (5)	0.0234 (4)	0.0176 (4)	0.0038 (4)	−0.0022 (3)	0.0019 (3)
C24	0.0208 (4)	0.0269 (5)	0.0213 (4)	0.0036 (4)	−0.0047 (3)	−0.0001 (4)
C25	0.0173 (4)	0.0235 (4)	0.0201 (4)	0.0008 (3)	−0.0014 (3)	−0.0009 (3)
C26	0.0170 (4)	0.0172 (4)	0.0154 (4)	0.0009 (3)	0.0000 (3)	−0.0013 (3)

### Geometric parameters (Å, º)

**Table:**

C1—C6	1.4014 (13)	C13—C14	1.4129 (16)
C1—C2	1.4022 (12)	C13—H13	1.005 (14)
C1—C8	1.4850 (12)	C14—C15	1.3729 (14)
C2—C3	1.3995 (12)	C14—H14	0.999 (13)
C2—H2	0.968 (13)	C15—C16	1.4224 (13)
C3—C4	1.3999 (13)	C15—H15	0.970 (14)
C3—C18	1.4856 (12)	C17—C18	1.3830 (12)
C4—C5	1.3922 (13)	C17—C26	1.4190 (12)
C4—H4	1.005 (13)	C17—H17	1.000 (13)
C5—C6	1.3907 (13)	C18—C19	1.4243 (12)
C5—H5	0.959 (14)	C19—C20	1.3707 (12)
C6—H6	0.989 (13)	C19—H19	1.012 (13)
C7—C8	1.3829 (12)	C20—C21	1.4193 (12)
C7—C16	1.4193 (13)	C20—H20	0.990 (13)
C7—H7	0.991 (13)	C21—C22	1.4168 (12)
C8—C9	1.4250 (13)	C21—C26	1.4204 (12)
C9—C10	1.3698 (13)	C22—C23	1.3702 (14)
C9—H9	0.997 (13)	C22—H22	0.994 (13)
C10—C11	1.4226 (13)	C23—C24	1.4136 (14)
C10—H10	1.006 (13)	C23—H23	1.020 (14)
C11—C12	1.4178 (13)	C24—C25	1.3731 (13)
C11—C16	1.4217 (13)	C24—H24	0.992 (13)
C12—C13	1.3723 (14)	C25—C26	1.4229 (13)
C12—H12	1.010 (13)	C25—H25	0.987 (13)
			
C6—C1—C2	118.39 (8)	C15—C14—C13	120.59 (9)
C6—C1—C8	121.35 (8)	C15—C14—H14	118.9 (8)
C2—C1—C8	120.25 (8)	C13—C14—H14	120.4 (8)
C3—C2—C1	121.85 (8)	C14—C15—C16	120.54 (9)
C3—C2—H2	119.4 (7)	C14—C15—H15	121.1 (8)
C1—C2—H2	118.7 (7)	C16—C15—H15	118.4 (8)
C2—C3—C4	118.50 (8)	C7—C16—C11	119.27 (8)
C2—C3—C18	120.41 (8)	C7—C16—C15	121.95 (9)
C4—C3—C18	121.08 (8)	C11—C16—C15	118.78 (8)
C5—C4—C3	120.28 (8)	C18—C17—C26	121.30 (8)
C5—C4—H4	120.1 (7)	C18—C17—H17	121.9 (7)
C3—C4—H4	119.7 (7)	C26—C17—H17	116.7 (7)
C6—C5—C4	120.68 (9)	C17—C18—C19	119.01 (8)
C6—C5—H5	120.4 (8)	C17—C18—C3	121.10 (8)
C4—C5—H5	119.0 (8)	C19—C18—C3	119.88 (8)
C5—C6—C1	120.28 (8)	C20—C19—C18	120.78 (8)
C5—C6—H6	120.0 (8)	C20—C19—H19	118.9 (7)
C1—C6—H6	119.7 (8)	C18—C19—H19	120.3 (7)
C8—C7—C16	121.51 (9)	C19—C20—C21	120.87 (8)
C8—C7—H7	120.6 (7)	C19—C20—H20	120.2 (7)
C16—C7—H7	117.9 (7)	C21—C20—H20	118.9 (7)
C7—C8—C9	118.52 (8)	C22—C21—C20	121.90 (8)
C7—C8—C1	121.48 (8)	C22—C21—C26	119.12 (8)
C9—C8—C1	120.00 (8)	C20—C21—C26	118.96 (8)
C10—C9—C8	121.31 (8)	C23—C22—C21	120.80 (9)
C10—C9—H9	120.3 (8)	C23—C22—H22	122.1 (7)
C8—C9—H9	118.4 (8)	C21—C22—H22	117.1 (7)
C9—C10—C11	120.75 (9)	C22—C23—C24	120.30 (9)
C9—C10—H10	121.1 (7)	C22—C23—H23	119.3 (7)
C11—C10—H10	118.1 (7)	C24—C23—H23	120.4 (7)
C12—C11—C16	119.28 (8)	C25—C24—C23	120.29 (9)
C12—C11—C10	122.08 (9)	C25—C24—H24	119.8 (8)
C16—C11—C10	118.64 (8)	C23—C24—H24	119.9 (8)
C13—C12—C11	120.71 (9)	C24—C25—C26	120.60 (9)
C13—C12—H12	120.2 (8)	C24—C25—H25	120.5 (8)
C11—C12—H12	119.1 (8)	C26—C25—H25	118.8 (8)
C12—C13—C14	120.10 (9)	C17—C26—C21	119.06 (8)
C12—C13—H13	119.4 (8)	C17—C26—C25	122.01 (8)
C14—C13—H13	120.4 (8)	C21—C26—C25	118.89 (8)
			
C6—C1—C2—C3	1.51 (13)	C10—C11—C16—C7	−0.07 (13)
C8—C1—C2—C3	−177.83 (8)	C12—C11—C16—C15	−0.31 (13)
C1—C2—C3—C4	−1.46 (13)	C10—C11—C16—C15	179.66 (9)
C1—C2—C3—C18	177.73 (8)	C14—C15—C16—C7	179.43 (9)
C2—C3—C4—C5	0.54 (13)	C14—C15—C16—C11	−0.28 (15)
C18—C3—C4—C5	−178.65 (9)	C26—C17—C18—C19	−0.81 (14)
C3—C4—C5—C6	0.30 (14)	C26—C17—C18—C3	178.19 (8)
C4—C5—C6—C1	−0.24 (15)	C2—C3—C18—C17	−145.04 (9)
C2—C1—C6—C5	−0.64 (14)	C4—C3—C18—C17	34.14 (13)
C8—C1—C6—C5	178.69 (9)	C2—C3—C18—C19	33.95 (12)
C16—C7—C8—C9	0.27 (14)	C4—C3—C18—C19	−146.87 (9)
C16—C7—C8—C1	−179.14 (8)	C17—C18—C19—C20	−0.30 (14)
C6—C1—C8—C7	−36.04 (13)	C3—C18—C19—C20	−179.31 (8)
C2—C1—C8—C7	143.28 (9)	C18—C19—C20—C21	1.32 (14)
C6—C1—C8—C9	144.56 (9)	C19—C20—C21—C22	177.04 (9)
C2—C1—C8—C9	−36.12 (13)	C19—C20—C21—C26	−1.24 (14)
C7—C8—C9—C10	0.22 (14)	C20—C21—C22—C23	−177.55 (9)
C1—C8—C9—C10	179.64 (9)	C26—C21—C22—C23	0.72 (14)
C8—C9—C10—C11	−0.64 (15)	C21—C22—C23—C24	−0.75 (15)
C9—C10—C11—C12	−179.48 (9)	C22—C23—C24—C25	0.27 (15)
C9—C10—C11—C16	0.55 (14)	C23—C24—C25—C26	0.23 (15)
C16—C11—C12—C13	0.59 (14)	C18—C17—C26—C21	0.87 (13)
C10—C11—C12—C13	−179.38 (9)	C18—C17—C26—C25	−177.02 (9)
C11—C12—C13—C14	−0.27 (15)	C22—C21—C26—C17	−178.18 (8)
C12—C13—C14—C15	−0.34 (16)	C20—C21—C26—C17	0.15 (13)
C13—C14—C15—C16	0.61 (16)	C22—C21—C26—C25	−0.22 (13)
C8—C7—C16—C11	−0.34 (14)	C20—C21—C26—C25	178.10 (8)
C8—C7—C16—C15	179.94 (9)	C24—C25—C26—C17	177.64 (9)
C12—C11—C16—C7	179.96 (9)	C24—C25—C26—C21	−0.25 (14)
